# OFDMA Backoff Control Scheme for Improving Channel Efficiency in the Dynamic Network Environment of IEEE 802.11ax WLANs

**DOI:** 10.3390/s21155111

**Published:** 2021-07-28

**Authors:** Youngboo Kim, Lam Kwon, Eun-Chan Park

**Affiliations:** Department of Information and Communication Engineering, Dongguk University-Seoul, Seoul 04620, Korea; 0bookim@dongguk.edu (Y.K.); lamk@dongguk.edu (L.K.)

**Keywords:** UORA, OFDMA backoff control, IEEE 802.11ax, WLAN

## Abstract

IEEE 802.11ax uplink orthogonal frequency division multiple access (OFDMA)-based random access (UORA) is a new feature for random channel access in wireless local area networks (WLANs). Similar to the legacy random access scheme in WLANs, UORA performs the OFDMA backoff (OBO) procedure to access the channel and decides on a random OBO counter within the OFDMA contention window (OCW) value. An access point (AP) can determine the OCW range and inform each station (STA) of it. However, how to determine a reasonable OCW range is beyond the scope of the IEEE 802.11ax standard. The OCW range is crucial to the UORA performance, and it primarily depends on the number of contending STAs, but it is challenging for the AP to accurately and quickly estimate or keep track of the number of contending STAs without the aid of a specific signaling mechanism. In addition, the one for this purpose incurs an additional delay and overhead in the channel access procedure. Therefore, the performance of a UORA scheme can be degraded by an improper OCW range, especially when the number of contending STAs changes dynamically. We first observed the effect of OCW values on channel efficiency and derived its optimal value from an analytical model. Next, we proposed a simple yet effective OBO control scheme where each STA determines its own OBO counter in a distributed manner rather than adjusting the OCW value globally. In the proposed scheme, each STA determines an appropriate OBO counter depending on whether the previous transmission was successful or not so that collisions can be mitigated without leaving OFDMA resource units unnecessarily idle. The results of a simulation study confirm that the throughput of the proposed scheme is comparable to the optimal OCW-based scheme and is improved by up to 15 times compared to the standard UORA scheme.

## 1. Introduction

Nowadays, the Internet of Things (IoT) is being used in various fields and expanding its applicable scope to various areas such as context-aware intelligent services [1], protected agriculture [2], healthcare [3], and public safety [4]. In other words, if the IoT environment will become commonplace in the near future, a massive number of wireless devices will be concentrated in a narrow area, so the performance of wireless communication systems might degrade. This issue should also be addressed in other systems in which a wireless communication system is required, such as crowd sensing [5] and video surveillance [6] in smart cities, elderly healthcare [7] in smart homes, and intelligent transportation systems [8] in the future traffic system.

Meanwhile, wireless local area networks (WLANs) are still effective for supporting IoT or smart city environments among various wireless communication systems thanks to their low deployment cost and high throughput.

In addition, the IEEE 802.11ax [9], the most up-to-date WLAN standard, is designed to solve the performance degradation problem, which occurs in an environment where many access points (APs) and stations (STAs) coexist. Thus, a future WLAN system will be able to manage a massive number of IoT devices effectively [10,11,12,13].

In IEEE 802.11ax, a significant change to deal with many APs and STAs is the introduction of orthogonal frequency division multiple access (OFDMA) for supporting multi-user transmission.

Although random access schemes such as distributed coordination function (DCF) or enhanced distributed channel access (EDCA) are used to occupy or share radio resources in the previous WLAN standard, they are not applicable to the OFDMA system. Therefore, uplink OFDMA-based random access (UORA), which is a new feature for random channel access in OFDMA-based WLANs, has been introduced in IEEE 802.11ax.

In the UORA mechanism, the channel is divided into several sub-carrier groups referred to as resource units (RUs). These comprise the minimum unit for an OFDMA resource with which an STA can access the channel and transmit a frame. Multiple STAs can transmit data frames at the same time with different RUs. For the operation of multi-user transmission, UORA introduces the OFDMA contention window (OCW) and OFDMA backoff (OBO) counter. To transmit a frame, each STA selects a random OBO counter within the OCW value and decreases it by the number of RUs available for UORA. If the decreased OBO counter becomes less than or equal to zero, the STA is allowed to transmit the frame with an arbitrarily available RU (the detailed operation of UORA is described in Section 2.1). Similar to DCF or EDCA, the performance of UORA is very sensitive to the number of contending STAs and the OCW range. Therefore, in UORA, an AP has a crucial role in determining the proper range of OCW and informing the STAs of this. In contrast to DCF and EDCA, where the range of contention window (CW) is pre-determined, UORA can flexibly control the OCW range depending on the number of contending STAs.

However, it is difficult for the AP to determine the appropriate OCW range in practice because a mobile STA frequently joins or leaves a basic service set (BSS), thereby making it impossible for the AP to estimate the transmission buffer status of each STA exactly without a dedicated signaling mechanism of *buffer status report* (BSR). In other words, it is difficult for the AP to know the exact number of STAs contending to access RUs in each UORA procedure because it changes dynamically. Even with BSR signaling, the control frame containing the BSR information is not always successfully delivered due to collisions. Furthermore, the BSR signaling incurs an additional delay and overhead in the channel access, which is not desirable in dense WLANs with many STAs.

This study aims to maximize the efficiency of UORA by decreasing RU collisions or idle RUs. We first observed the effect of the OCW value and derived its optimal value for maximizing the channel efficiency. We discovered an interesting result in that the transmission collision probability of STAs is almost immune to the number of contending STAs if all of them maintain the optimal OCW value. From this observation, we propose an OBO control scheme that operates in a distributed way without requiring the determination of the optimal OCW value. In our proposed scheme, each STA controls its OBO counter adaptively according to the result of the previous transmission (failure or success). Once the transmission is made successfully, the proposed scheme decreases the OBO value rapidly so that the STA can access the channel in a more aggressive manner and the number of idle RUs can be decreased. Otherwise, if the previous transmission fails, the OBO value decreases slowly to avoid severe collisions.

The rest of this paper is organized as follows. In Section 2, we describe the details of the IEEE 802.11ax UORA scheme and related work in the literature. Moreover, we observe the effect of OCW and derive its optimal value from an analytical model in Section 3 and describe our proposed scheme in Section 4. In Section 5, we evaluate and compare the performance of the proposed mechanism from the results of simulations and, finally, conclude the paper in Section 6.

## 2. Background

### 2.1. Uplink OFDMA Random Access (UORA)

**Figure 1:**
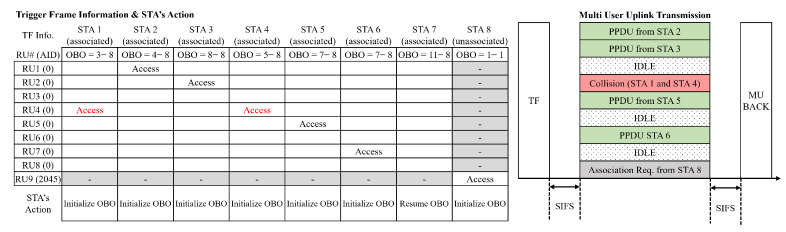
An example of a UORA operation in the IEEE 802.11ax standard.

Two different types of uplink multi-user (MU) OFDMA operations, scheduled access and random access, are defined in the IEEE 802.11ax standard [9]. In scheduled access, the STAs share the OFDMA RUs in a contention-free manner, and each STA requests the transmission permission to the AP by means of BSR signaling. Subsequently, the AP allocates a dedicated RU to a specific STA by transmitting a trigger frame (TF) containing the scheduling information. On the other hand, in a random access mode, the STA acquires the RU in a contention-based manner according to the UORA mechanism illustrated in Figure 1.

In the UORA mechanism, the AP first sends a TF to initiate the UORA procedure. The TF contains several pieces of information, such as the eligible random access RUs (RA-RUs) and the corresponding association identifiers (AIDs). In the 802.11ax standard, the AID of RA-RUs is either 0 or 2045: RA-RUs with an AID of 0 can be accessed by the associated STAs, whereas the unassociated STAs can occupy RA-RUs with an AID of 2045. It is possible that AP can allocate some RUs for scheduled access and others for random access. However, to focus on the performance of UORA, we assume that all of the RUs are eligible for random access without considering the scheduled access. More specifically, we set one RU to have an AID of 2045 and set all of the remaining with an AID of 0. The total number of RUs depends on the channel bandwidth and the number of sub-carriers per RU, as specified in the IEEE 802.11ax standard.

After receiving the TF, each STA determines its OBO counter based on the OCW range advertised by the AP. The initial OBO counter is a random positive integer that is uniformly distributed within the OCW range. Next, an associated or unassociated STA decreases its OBO counter by the number of RUs with an AID of 0 or 2045, respectively. After updating the OBO counter, the STA attempts to access an arbitrary RU only if its OBO counter is less than or equal to 0. However, a transmission collision can occur if two or more STAs access the same RU. The AP informs each STA whether or not the transmission is successful by using multi-user block acknowledgment (MU-BACK). In each STA, the initial OCW value is $OCW_{min}$, and the OCW value is doubled whenever the transmission fails. The OCW cannot exceed $OCW_{max}$ and is reset to $OCW_{min}$ when the transmission succeeds. Note that this OCW control operation, which is called binary exponential backoff (BEB), is identical to that of CW control in DCF and EDCA.

Meanwhile, the AP can configure and advertise the OCW range ($OCW_{min}$ and $OCW_{max}$) by broadcasting management frames such as beacon or fast initial link setup discovery frames. The OCW range can also be contained in several unicast management frames (e.g., probe response, association response, and re-association response frames). These various management frames contain two 3-bit OCW range fields, EOCWmin and EOCWmax. On receiving these frames, the STA sets $OCW_{min}=2^{\mathrm{EOCWmin}}-1$ and $OCW_{max}=2^{\mathrm{EOCWmax}}-1$. If the STA does not receive the OCW range field from the AP, it uses the default OCW range (i.e., $OCW_{min}$ = 7 and $OCW_{max}$ = 31).

Figure 1 depicts an example of UORA operation. Here, STAs 1–8 are associated, and STA 9 is unassociated. We consider that the channel bandwidth is 20 MHz, and there are nine RA-RUs consisting of eight RUs with AID 0 and one with AID 2045. Thus, on receiving the TF, STA 1–8 decrease their OBO counters by 8, and STA 9 decreases its OBO counter by 1. In this example, the OBO counters of STA 1–6 become ≤0, and so they select a random RA-RU among RU 1–8 to transmit a frame. However, since STA 7 maintains its OBO counter greater than 0, it cannot access the channel and so decreases its OBO counter upon receiving the next TF. It is worth noting that the RU can either collide or remain idle. For example, both STA 1 and STA 4 access RU 4, so their transmissions can fail due to RU collision. On the other hand, some of the RUs (3, 6, and 8) are not accessed by any of the STAs and so are wasted. To maximize the channel efficiency, the numbers of colliding and idle RUs should be minimized, which is difficult to achieve due to the nature of the distributed and random operation of UORA. It is also noteworthy that the backoff procedure for UORA is different from that of DCF or EDCA. While the latter two perform the backoff procedure in the time domain to determine when to transmit, that of UORA is two-dimensional, i.e., it determines which RU to occupy in the frequency domain and, at the same time, establishes the transmission time.

### 2.2. Related Work

Several studies [14,15,16,17,18] have proposed performance analysis methods for UORA based on a two-dimensional Markov chain model [19]. At the early stage of IEEE 802.11ax standardization, the authors in [14] established an analytic model to evaluate the throughput of UORA with which the optimal number of RA-RUs can be obtained to maximize the throughput. Similar to [14], another analytic model for UORA was proposed by [15]. As well as providing models of throughput and channel efficiency, a throughput optimization algorithm was proposed under the assumption that the AP can estimate the number of STAs. After the detailed procedure of UORA had been specified in the IEEE 802.11ax standard, a new analytic model was designed by [16] that reflected the updated operation of UORA, including the change in OBO decrement rule. This model was used to analyze the performance of UORA in terms of the system efficiency and average access delay. The authors in [17] considered the case where both random access and scheduled access are combined for uplink transmission in IEEE 802.11ax WLANs: the BSR frame is transmitted according to the UORA mechanism, whereas the data frame is transmitted by means of scheduled access. They investigated the trade-off between increasing the network throughput and supporting new STAs by defining a new performance index named the BSR delivery rate.

In [18], the author provided an analytical framework for the 802.11ax MAC protocol considering both non-saturated traffic conditions and co-existence with the legacy nodes.

In the literature, various approaches have been proposed to improve the performance of UORA [20,21,22]. In [20], a scheme named *Hybrid Uplink OFDMA Random Access* (H-UORA) was proposed. To reduce transmission collisions, H-UORA introduces an RU-sensing slot for additional channel sensing. Similar to H-UORA, the aim of the *Collision Reduction and Utilization Improvement* (CURI) mechanism in [21] is to decrease transmission collisions. CURI consists of two schemes, extra backoff (EBO) and opportunistic RU hopping (ORH): in the former, each STA sends a busy signal according to the priority of STA before transmitting data, while the latter is used to improve channel efficiency to provide a second opportunity for RU access by an STA that could not occupy any of the RUs during the EBO stage. The mechanism proposed in [22] provides a way to reduce collisions based on the virtual time slot (VTS) in multi-user multiple input and multiple output (MU-MIMO)-enabled UORA; collisions can be avoided with this mechanism by differentiating VTSs (i.e., the starting time of data transmission) in STAs.

The studies in [23,24,25,26] were focused on underlying the drawbacks of the BEB mechanism in OCW control. In [23], the *Retransmission Number Aware Channel Access* (RNACA) scheme was proposed to avoid the increase of transmission delay due to collisions. By considering the number of retransmissions, the number of RUs, and the number of contending STAs, RNACA doubles the OCW value in a probabilistic way so that some of the STAs can access the RU without doubling the OCW value. The approach in [24] deals with the problem of increased delay due to the doubled OCW value. To solve this problem, the authors proposed the *probability complementary transmission scheme* (PCTS), with which the STA performs complementary transmission without backoff. Moreover, the authors in [25,26] considered the problem of BEB in highly dense WLANs. Instead of blindly doubling/resetting the CW on the transmission failure/success, respectively, the backoff mechanism proposed in [25], *Channel Observation-based Scaled Backoff* (COSB), adaptively increases/decreases the CW depending on the estimated collision probability. The same authors in [25] further enhanced the performance of COSB by utilizing the Q-learning (QL) model [26], which is one of the prevailing deep reinforcement learning technologies. The mechanism named *intelligent QL-based resource allocation* (iQRA) scales the CW to maximize the established reward in the QL model.

Meanwhile, the authors in [27,28,29] attempted to combine UORA with other features in IEEE 802.11ax to improve the channel efficiency. In [27], the target wake time (TWT) mechanism, aimed at improving the power-saving performance of IEEE 802.11ax, was considered for grouping the STAs. By employing TWT, the STAs are classified into different groups, and their wake and sleep times are controlled collectively. The number of STAs contending to access RUs can be controlled by means of TWT, and thus transmission collisions can be decreased. In [27], the optimal number of STAs in a group was also derived to maximize the channel efficiency. This work was extended in [28] by considering a network situation where the STA delivers BSR based on UORA; the relationship between group size and RU efficiency was analyzed, and an adaptive grouping algorithm with variable group size was proposed to achieve the optimal efficiency of BSR delivery. The authors in [29] proposed *Multi-dimensional Busy-Tone Arbitration* (MBTA) to decrease the number of collisions during BSR transmission with UORA. They also designed *Dynamic Access Mode Selection* (DAMS) with which APs or STAs can determine the optimal access mode: either random access or scheduled access.

Compared to these existing studies, our one has the following novelties and advantages:

- To the best of our knowledge, our study is the first approach to control the OBO counter for improving the performance of UORA. Instead of controlling OCW, our scheme controls the rate of OBO counter decrement in a distributed manner so that it can be considered as analogous to virtually adjusting the number of RUs.
- Our scheme does not require any control frame or additional signaling between the STA and the AP. It can be simply implemented with a minimal change in the STA while fully complying with the standard UORA scheme.
- Our scheme only changes the OBO control rule, so it does not conflict with the existing approaches and can be easily integrated into them to further improve the performance of UORA.

## 3. Analysis of the Optimal OFDMA Contention Window

We derive the optimal value of OCW that maximizes the channel efficiency of UORA and observe the effect of the OCW range ($OCW_{min}$ to $OCW_{max}$). For this purpose, we employ the analytic model provided in [16]. We define $N_{sta}$ and $M_{ru}$ as the number of associated STAs contending to access RA-RUs and the number of available RA-RUs with AID 0, respectively. To obtain the optimal value of OCW, $W^{*}$, we assume that both $N_{sta}$ and $M_{ru}$ are fixed and that the AP is aware of the exact value of $N_{sta}$.

Let us denote $p_{c}$ as the conditional probability that the transmitted frame with a certain RU collides with another frame transmitted with the same RU. Therefore, $p_{c}$ is equal to the probability that at least one of the ($N_{sta}-1$) remaining STAs transmits by using the selected RU, which can be represented as

(1) $$p_{c}=1-{\left(1-\frac{\tau }{M_{ru}}\right)}^{N_{sta}-1},$$

where τ is the probability that an STA transmits a frame at the given UORA channel access contention (i.e., its OBO counter is not greater than $M_{ru}$). According to the result in [16], τ can be expressed as

(2) $$\tau =\frac{W+1}{\left(1-p_{c}\right)\left(W+1+X\right)},$$

where

(3) $$X=\left(W-\frac{M_{ru}}{2}\right)\lfloor \frac{W}{M_{ru}}\rfloor -\frac{M_{ru}}{2}\lfloor \frac{W}{M_{ru}}\rfloor^{2}.$$

and *W* denotes the value of OCW. We intentionally disabled the BEB mechanism to obtain the optimal value of OCW (i.e., $W=OCW_{min}=OCW_{max}$). Note that in (1) and (2), the collision probability $p_{c}$ and access probability τ are mutually related to each other and can be numerically calculated. Next, we define the channel efficiency μ as the ratio of the expected number of STAs that successfully transmit a frame to the number of RUs, which is equivalent to the fraction of RUs that are neither idle nor colliding. From (1) and (2), μ can be expressed as

(4) $$\mu =\frac{N_{sta}\tau \left(1-p_{c}\right)}{M_{ru}}.$$

**Figure 2:**
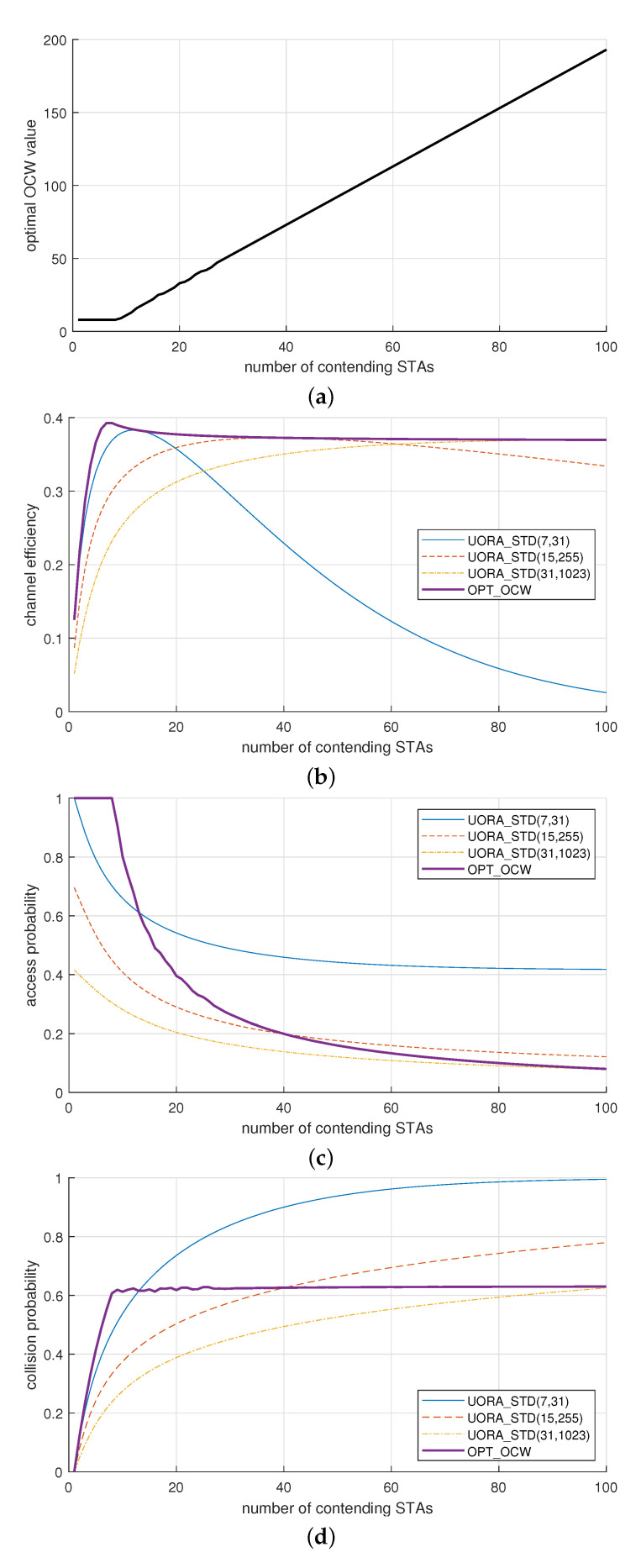
Analysis of the OCW effect in UORA: (**a**) the optimal OCW value, (**b**) channel efficiency, (**c**) access probability, and (**d**) collision probability.

From (1)–(4), we can numerically obtain the optimal OCW value $W^{*}$ maximizing μ when $N_{sta}$ and $M_{ru}$ are given. Figure 2a shows $W^{*}$ with respect to $N_{sta}$ ($1\leq N_{sta}\leq 100$) when $M_{ru}$ is 8. We can observe from Figure 2a that $W^{*}$ increases almost linearly as long as $N_{sta}>M_{ru}$, which is the same as in conventional DCF [19].

We can observe the effect of OCW when the BEB mechanism is enabled, as standardized in IEEE 802.11ax. In addition, we can compare the UORA scheme with the optimal OCW and standard UORA schemes with the BEB mechanism where the minimum and maximum values of OCW are $OCW_{min}$ and $OCW_{max}$, respectively. We denote the former as OPT_OCW and the latter as $\mathrm{UORA}\_\mathrm{STD}(OCW_{min},OCW_{max})$. We can observe the performance in terms of the channel efficiency, access probability, and collision probability, which can be obtained from (1)–(4). To consider the BEB mechanism, we need to revise the access probability τ in (2) as described in [16]. As shown in Figure 2b, the channel efficiency of OPT_OCW increases to the maximum value of 0.38 when $N_{sta}$ increases up to $M_{ru}$ (=8), and it is almost constant as long as $N_{sta}>M_{ru}$. It is worth noting that the maximum efficiency of UORA even with the optimal value of OCW does not exceed 0.4, which is in agreement with the literature [14,15,16]. This result is similar to slotted ALOHA and mainly stems from the nature of random access. Poor maximum efficiency is inevitable in distributed and contention-based random access, and the efficiency can be improved by centralized and scheduling-based access.

On the other hand, in the case of UORA_STD, although the maximum channel efficiency can be attained with a specific value of $N_{sta}$ and is comparable to that in OPT_OCW, it is remarkably affected by the value of $N_{sta}$ and much lower than the maximum value. For example, in the case of UORA_STD(7,31), the efficiency was smaller than 0.25 when $N_{sta}>40$ whereas it was greater than 0.35 for the same range of $N_{sta}$ in the cases of UORA_STD(15,255) and UORA_STD(31,1023). The result in Figure 2b confirms that the performance of UORA with the default OCW range (i.e., ($OCW_{min}$, $OCW_{max}$) = (7,31)) may be significantly degraded as $N_{sta}$ exceeds a certain value. Although this problem can be mitigated by a larger OCW range (e.g., $\left(OCW_{min},OCW_{max}\right)=\left(15,255\right)$ or (31,1023)), this configuration rather decreases the efficiency when $N_{sta}$ has a small value.

Figure 2c shows that, compared to UORA_STD, OPT_OCW accesses the channel more aggressively or conservatively when $N_{sta}$ is small (<20) or large (>50), respectively. We can observe an interesting result in Figure 2d; as long as $N_{sta}>M_{ru}$, the collision probability of UORA_STD increases with respect to *n*, whereas that of OPT_OCW is almost constant regardless of $N_{sta}$.

## 4. The OFDMA Backoff Control Scheme

### 4.1. Design Rationale and Requirement

The results of the analysis in Section 3 imply that the performance of UORA can be greatly improved if the AP can exactly estimate $N_{sta}$ and instantly inform the STAs about the proper values of $OCW_{min}$ and $OCW_{max}$. However, this approach cannot be simply realized without the aid of BSR signaling. Recall that $N_{sta}$ is the number of STAs that participate in the contention at a given time rather than the number of STAs that are associated with the AP; thus, $N_{sta}$ can change dynamically. Since $N_{sta}$ is the number of STAs in which the transmission buffer is not empty, BSR signaling is essential to estimate $N_{sta}$ accurately. The IEEE 802.11ax standard specifies two methods for BSR signaling, *solicited BSR* and *unsolicited BSR*. In response to a TF containing a *buffer status report poll* (BSRP), the STA can explicitly deliver the BSR information via UORA. Delivery of the BSR can fail due to a collision in UORA or be delayed due to insufficient RA-RUs. Moreover, a corresponding ACK is also required for the solicited BSR delivery. This approach inevitably increases signaling delay and the overhead, leading to a decrease in channel efficiency. As a result, it is neither desirable nor effective in dense WLANs where many STAs compete for channel access and multiple BSSs overlap. The STA can also deliver BSR in an unsolicited way by piggybacking it in the frame destined to the AP. Thus, the BSR signaling overhead can be decreased, although the unsolicited BSR delivery is not always available. For example, if an STA has just woken up from power-saving mode or the STA’s buffer has become empty, the BSR cannot be delivered in this way. Due to these reasons, we considered the BSR-free UORA in this study.

We can consider two approaches to deal with RU collisions: OCW control and OBO control. The conventional mechanism for OCW control is BEB, as adopted in the standard, and several solutions have been proposed to overcome the drawback of BEB [23,24,25,26]. Instead of OCW control, we considered a novel approach for OBO control due to the following two reasons. First, the performance with OCW control is greatly affected by the number of contending STAs, as already confirmed in Figure 2. Second, the IEEE 802.11ax standard mandates default values for $OCW_{min}$ and $OCW_{max}$. If the AP cannot advertise the OCW range in a timely and proper manner, the STAs should abide by these default values.

By considering these issues comprehensively, we tried to improve the performance of UORA and set the requirement of our approach as follows;

- To avoid the signaling overhead and delay due to BSR, we designed a distributed control scheme without resorting to the information about the number of contending STAs.
- The performance of the proposed OBO control should be comparable to the OCW control with the optimal value and as robust as possible to changes in the number of contending STAs.
- The proposed mechanism needs to be compatible with the standard UORA scheme for which the BEB mechanism in OCW control is mandated. Thus, we attempted to modify the OBO calculation procedure while maintaining the same OCW values as the standard.

### 4.2. Proposed OFDMA Backoff Control

We can infer from Figure 2c,d that we can improve the performance of UORA_STD by making the transmission attempt more aggressive or conservative (i.e., by allowing more or alleviating collisions) when $N_{sta}$ is small or large, respectively. To achieve this, we designed the OBO control such that the access probability is increased or decreased when the previous transmission succeeded or failed, respectively. This idea can be easily and practically realized by introducing a self-tunable parameter α in the OBO counter calculation. The idea of the proposed OBO control can be simply represented as

(5) $$OBO=OBO-\alpha \times M_{ru},$$

where

(6) $$\begin{array}{cc} \alpha & =\left\{\begin{array}{cc} min(\alpha +\delta ,\alpha_{max}), & \;\;\mathrm{on}\;\mathrm{transmission}\;\mathrm{success},\\ max(\alpha -\delta ,\alpha_{min}), & \;\;\mathrm{on}\;\mathrm{transmission}\;\mathrm{failure}. \end{array}\right. \end{array}$$

We set the initial value of α in (5) as 1. Note that the proposed OBO control scheme is not different from the standard UORA scheme at all if α is fixed to one; similarly, the former is equivalent to the latter when the number of available RUs is $\alpha \times M_{ru}$. In (6), δ(>0) is a fixed step to change the value of α, while $\alpha_{max}$ and $\alpha_{min}$ (0 < $\alpha_{min}\leq 1\leq \alpha_{max}$) are the maximum and minimum values of α, respectively.

Algorithm 1 presents the pseudo-code for the proposed scheme, including two comparative schemes as follows:

- UORA_STD: This is the standard UORA scheme where the OCW value changes according to the BEB mechanism.
- OPT_OCW: This scheme can maximize the efficiency of UORA by setting the OCW value as the optimal one $W^{*}$ calculated from (1)–(4) based on $N_{sta}$. Note that this is an ideal scheme but difficult to implement in practice because of the assumption that the AP is always aware of the exact value of $N_{sta}$ and immediately informs STAs of the change in $W^{*}$.
- OBO_CTRL: This is the proposed scheme.

The pseudo-code of these schemes comprises three different procedures: RECEIVE_TF(), ACK_TIMEOUT(), and RECEIVE_ACK().

The first procedure RECEIVE_TF() is invoked when the STA successfully receives a TF from the AP. In this procedure, the STA reads the number of RA-RUs ($M_{ru}$) from the TF. The operations UORA_STD and OPT_OCW are identical, i.e., the STA decreases the OBO counter by $M_{ru}$ informed in the TF. In OBO_CTRL, the decrement for the OBO counter is $\alpha M_{ru}$. If α> 1, the OBO quickly decreases and the STA is allowed to access the RU more aggressively. This is desirable when the number of contending STAs is small. Otherwise, if α< 1, the RU is accessed in a conservative manner, which contributes to a decrease in collision probability. The operations in the remaining part of RECEIVE_TF() comply with the IEEE 802.11ax standard.

The second procedure ACK_TIMEOUT() is called when the STA does not receive ACK within ack_timeout (i.e., the transmission fails). Note that the MU-BACK contains ACKs for the data frames successfully delivered to the AP. In this procedure, each scheme controls the OCW value to avoid a transmission collision at the next transmission. According to the IEEE 802.11ax standard, the OCW value is increased from OCW to 2×(OCW+1)−1 in UORA_STD and OBO_CTRL. Assuming that the transmission failure results from the collision, OBO_CTRL decreases the value of α by δ to decrease the collision probability. OPT_OCW sets its OCW as the optimal value calculated from $N_{sta}$ and $M_{ru}$.

The RECEIVE_ACK() procedure is performed when the transmission succeeds, i.e., the STA receives the corresponding ACK within ack_timeout. In this procedure, although both UORA_STD and OBO_CTRL initialize the OCW to the minimum value $OCW_{min}$, OBO_CTRL increases the value of α by δ to increase the transmission opportunity. In the case of OPT_OCW, the STA updates its optimal OCW value, which is the same as in ACK_TIMEOUT().

As can be seen in the pseudo-code, the proposed mechanism OBO_CTRL involves minimal feasible modification of UORA_STD by introducing the control parameter α. This tiny change led to a drastic performance improvement that was confirmed via simulations.
**Algorithm 1**: procedures for the three UORA schemes**procedure**Receive_TF()      Read the value of $M_{ru}$      **switch** Scheme type **do**        **case** UORA_STD or OPT_OCW           $OBO=OBO-M_{ru}$                **case** OBO_CTRL           $OBO=OBO-\alpha \times M_{ru}$            **end**    **if** OBO≤0 **then**        Access a random RU    **else**        Wait for the next trigger frame    **end** **end** **procedure**Ack_Timeout()    **switch** Scheme type **do**        **case** UORA_STD           OCW=2×(OCW+1)−1           $OCW=min(OCW,OCW_{max})$        **case** OPT_OCW           $OCW=Get\_Opt\_OCW(M_{ru},N_{sta})$        **case** OBO_CTRL           OCW=2×(OCW+1)−1           $OCW=min(OCW,OCW_{max})$           α = α - δ           α = $max(\alpha ,\alpha_{min})$    **end**    Select a new random integer OBO (1≤ OBO ≤ OCW) **end** **procedure**
Receive_Ack()    **switch** Scheme type **do**        **case** UORA_STD           $OCW=OCW_{min}$        **case** OPT_OCW           $OCW=Get\_Opt\_OCW(M_{ru},N_{sta})$        **case** OBO_CTRL           $OCW=OCW_{min}$           α = α + δ           α = $min(\alpha ,\alpha_{max})$    **end**    Select a new random integer OBO (1≤ OBO ≤ OCW) **end**


## 5. Simulation Study

We compared and evaluated the proposed scheme’s performance with other UORA schemes such as UORA_STD and OPT_OCW. In Section 5.1 and Section 5.2, we cover the effect of the number of contending STAs and the performance under dynamic network conditions where the entry and exit of STAs occur swiftly, respectively. In Section 5.3, we focus on the proposed scheme (OBO_CTRL) and investigate the effect of its key parameters ($\alpha_{min}$, $\alpha_{max}$, and δ) on throughput and fairness. In Section 5.4, we report on an evaluation of the performances of UORA schemes when the number of RA-RUs varies.

We implemented the simulator with MATLAB by considering key features of the UORA mechanism standardized in IEEE 802.11ax such as the trigger frame, MU uplink transmission, MU-BACK, and association procedure. The simulator codes used in this paper are available in https://github.com/0BoOKim/UORA_OBO_CTRL, accessed on 28 July 2021. In order to focus on the performance of UORA, the simulation was performed under the following assumptions; (i) the channel is ideal, i.e., the transmission fails only due to RU collision, (ii) the AP allocates all the RUs for random access, (iii) all the STAs always compete for the RUs, and they have the same frame size, modulation and coding rate.

The configurations and parameters used in the simulation are reported in Table 1. The values of OBO_CTRL’s parameters: δ, $\alpha_{min}$, and $\alpha_{max}$, were set to 0.1, 0.1, and 2.0, respectively. These values were determined via simulation study in Section 5.3. The values of $OCW_{min}$ and $OCW_{max}$ in OBO_CTRL were set to 7 and 31, respectively, which are the default values specified in the IEEE 802.11ax standard. Meanwhile, the OCW of OPT_OCW was set to the optimal value $W^{*}$, which can be numerically obtained from (1)–(4). Note that the value of $W^{*}$ depends on $N_{sta}$ and $M_{ru}$ but does not change according to the BEB mechanism.

### 5.1. Performance Comparison with Respect to the Number of Contending Stations

**Figure 3:**
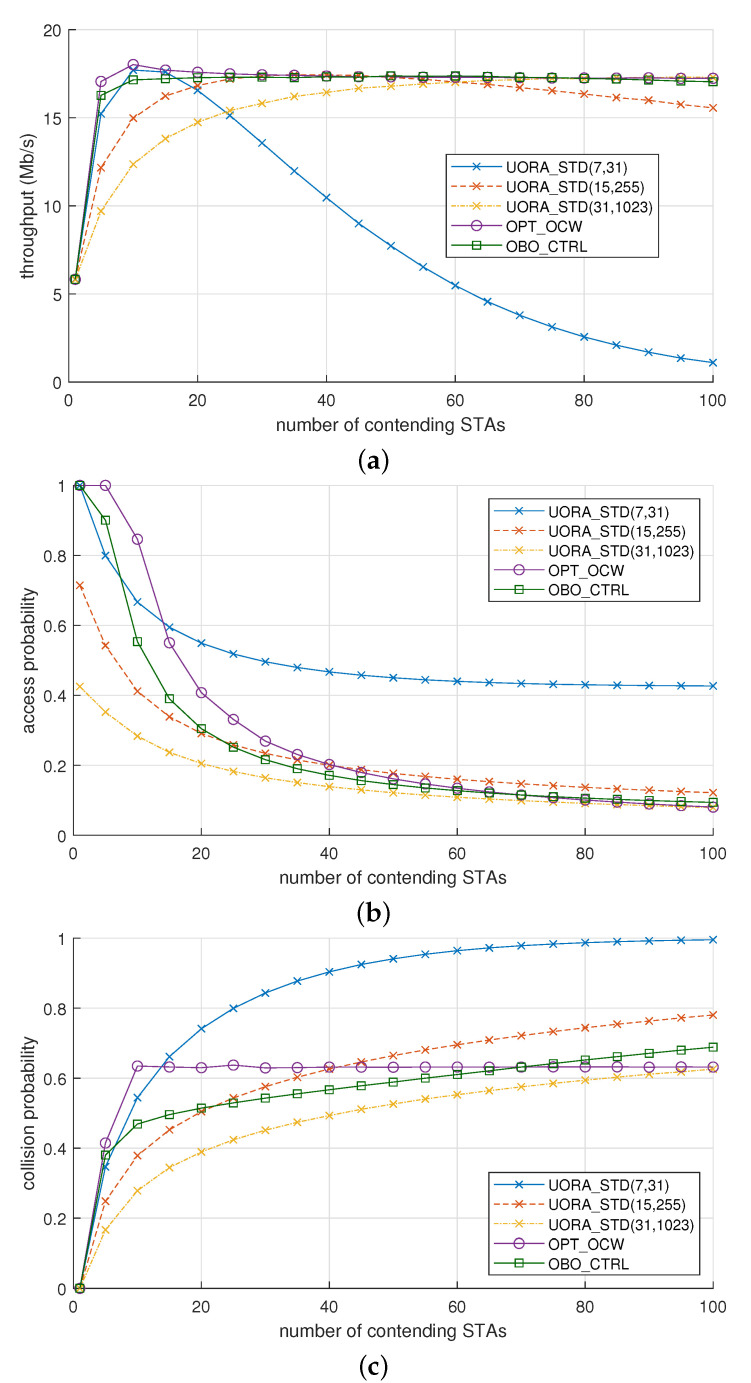
Performance comparison of the UORA schemes: (**a**) throughput, (**b**) access probability, and (**c**) collision probability.

Figure 3a shows the throughput of the UORA schemes when $N_{sta}$ was changed from 1 to 100. In UORA_STD(7,31), the throughput increased up to 17.7 Mb/s when $N_{sta}$ was increased from 1 to 10. However, as $N_{sta}$ was increased further from 15 to 100, the throughput decreased from 17.6 to 1.1 Mb/s. When the OCW range is increased and widened, the collision probability decreases when $N_{sta}$ is high, but the channel is more prone to being idle when $N_{sta}$ is low. As a result, compared to UORA_STD(7,31), UORA_STD(31,1023) achieved higher throughput when $N_{sta}>$ 30 but had lower throughput when $N_{sta}<$ 25. For UORA_STD(15,255), the throughput was between that of UORA_STD (7,31) and UORA_STD(31,1023). These results confirm the limitation of UORA_STD; the throughput can only be maximized for a specific value of $N_{sta}$ and is very sensitive to changes in $N_{sta}$. OPT_OCW and OBO_CTRL notably outperformed UORA_STD, with their throughputs being maintained at 17.1–18.0 and 16.3–17.4 Mb/s, respectively.

Figure 3b shows a comparison of the channel access probabilities of the UORA schemes. When $N_{sta}$ was low (1–10), the access probability of OBO_CTRL was higher than UORA_STD(15,255) and UORA_STD(31,1023) while comparable to UORA_STD(7,31). The higher access probability decreased the probability of idle RU, thereby contributing to the increase in throughput. As $N_{sta}$ increased, the access probability of OBO_CTRL decreased and gradually approached that of UORA_STD(31,1023). That is to say, OBO_CTRL restricted the excessive channel access, which led to higher throughput. Since OBO_CTRL differentiates the channel access probability depending on $N_{sta}$, it can maintain a sustained throughput regardless of the value of $N_{sta}$. Furthermore, as shown in Figure 3b, there was no significant difference between the access probabilities of OBO_CTRL and OPT_OCW. This result confirms that OBO_CTRL becomes comparable to OPT_OCW by simply controlling the OBO counter without using the optimal OCW value.

In Figure 3c, it can be observed that the collision probability for OPT_OCW was almost constant at around 0.63 as long as $N_{sta}>$ 10, which agrees well with the analysis result in Figure 2d. For OBO_CTRL, the collision probability increased from 0.47 to 0.69 when $N_{sta}$ increased from 10 to 100. The collision probability of OBO_CTRL was somewhat different from that of OPT_OCW but closer to the ideal OPT_OCW compared to the other schemes.

We can explain the reason why the collision probability of OBO_CTRL was less affected by $N_{sta}$ compared to UORA_STDs as follows. If $N_{sta}$ is small, OBO_CTRL allows STAs to access the RU in an aggressive way by increasing the value of α. Otherwise, if $N_{sta}$ is large, OBO_CTRL maintains a small value of α, which contributes to the decrease of collision due to excessive channel access. Later, it will be shown in Section 5.3 how OBO_CTRL controls the value of α depending on $N_{sta}$.

### 5.2. Performance Evaluation under Dynamic Network Environments

We investigated the performance of the UORA schemes when the number of contending STAs changes dynamically. Table 2 reports the results for five dynamic network scenarios (SCN_1 to SCN_5) considered in our simulations. Each scenario is characterized by four parameters $N_{sta}^{IN}$, $N_{sta}^{OUT}$, $T_{c}$, and $N_{sta}^{o}$; for every $T_{c}$ time, $N_{sta}^{IN}$ and $N_{sta}^{OUT}$ STAs join and leave the BSS, and $N_{sta}^{o}$ is the number of associated STAs in the BSS at the initial simulation time. Note that a new unassociated STA competes with other unassociated STAs for the RA-RU (AID = 2045) to perform the association procedure before joining the BSS. Once associated, the STA competes for the RA-RU (AID = 0) with existing associated STAs to transmit a data frame. The throughput was measured during every 100,000 slot time (0.9 s).

**Figure 4:**
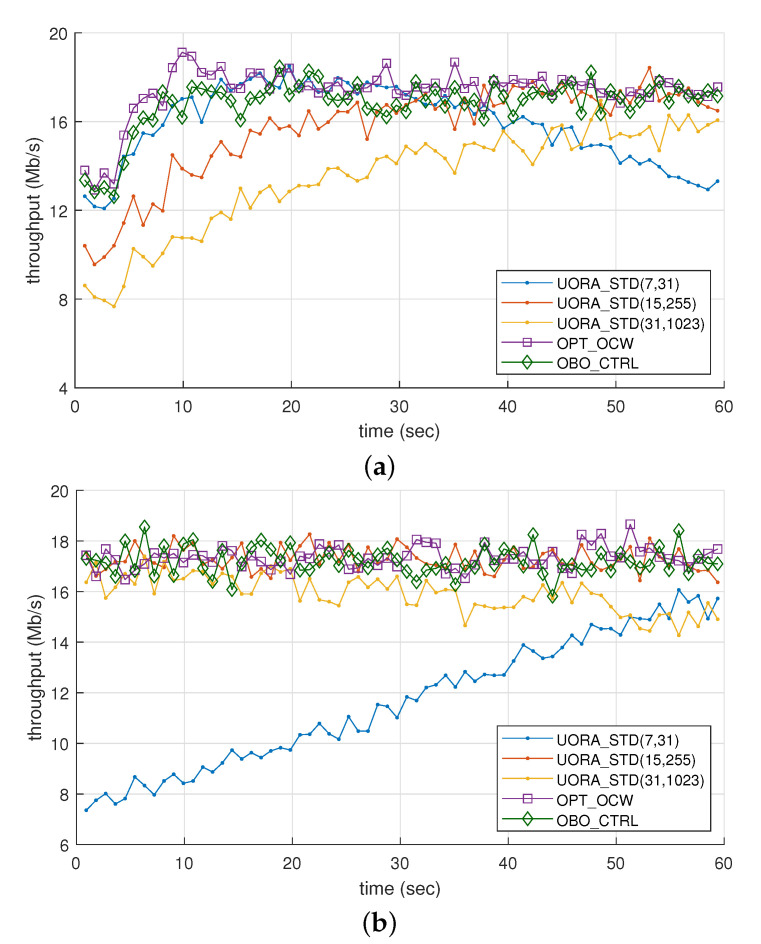
Throughput comparison of the UORA schemes under a dynamic network environment: (**a**) scenario 1 and (**b**) scenario 2.

Figure 4a shows throughput in SCN_1 where two STAs join every 4.0s (i.e., $N_{sta}^{IN}=2$ and $T_{c}=4.0$). In this scenario, up to 30 new STAs are associated until the end of the simulation. Recall that, as shown in Figure 3a, the throughput of UORA_STD(7,31) was maximized when $N_{sta}$ was around 10 and decreased when $N_{sta}$ exceeded 10, whereas the throughputs of other UORA_STD schemes increased gradually until $N_{sta}$ reached 30. These observations agree with the results in Figure 4a for the dynamic network scenario. Furthermore, we verified again that the throughput of OBO_CTRL was close to that of OPT_OCW even in the dynamic network environment, and it was hardly affected by the increase in the number of STAs.

Figure 4b shows the throughput in SCN_2 where the total number of STAs decreased from 50 to 20 so that the channel access contention was alleviated over time. The throughput of UORA_STD(7,31) almost linearly increased from 7.4 to 16.1 Mb/s during the whole simulation time, and that of UORA_STD(31,1023) rather slightly decreased from 16.4 to 14.3 after 30 s. On the other hand, the throughputs of OBO_CTRL and OPT_OCW were not changed notably over time, although the number of STAs gradually decreased. The results for the dynamic scenarios in Figure 4 match well with those for the static scenario in Figure 3a.

**Figure 5:**
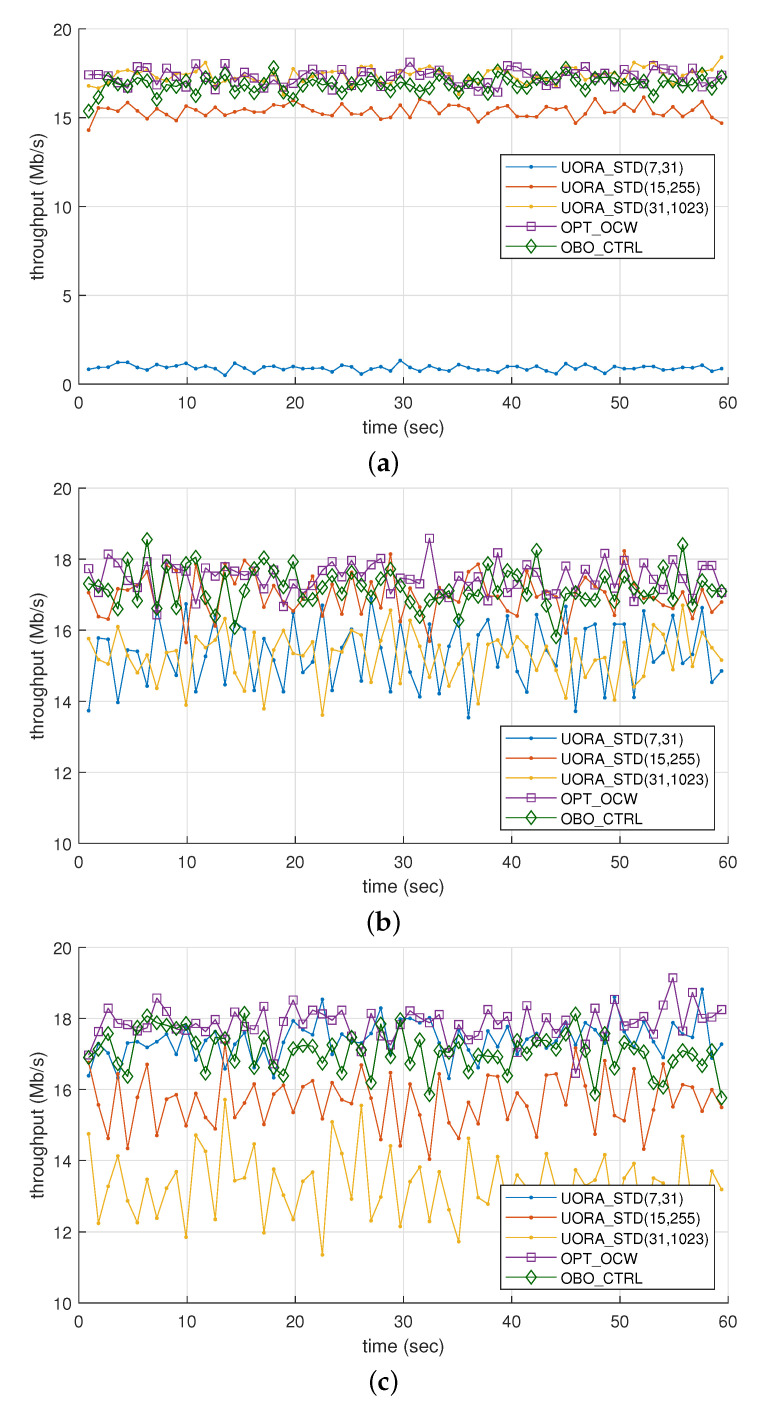
Throughput comparison of UORA schemes under dynamic network conditions: (**a**) scenario 3, (**b**) scenario 4, and (**c**) scenario 5.

Figure 5 shows how the throughput changes in response to fast changes in network conditions portrayed in SCN_3 to SCN_5, and Table 3 lists the average throughput and the differences between the 95th and 5th percentiles of the throughput of each UORA scheme (denoted as Δ). In the case of SCN_3 (see Figure 5a), UORA_STD(31,1023), OPT_OCW, and OBO_CTRL achieved similar throughput levels. However, the average throughput of UORA_STD(7,31) was significantly (more than 18 times) smaller than the other schemes.

In SCN_4 (Figure 5b), the throughput of all the schemes more fluctuated compared to SCN. Note that the scale of the Y-axis in Figure 5a is different from those in Figure 5b or Figure 5c. The variation of throughput can be evaluated with Δ given in Table 3. Meanwhile, OPT_OCW achieved the highest average throughput (17.48 Mb/s) and UORA_STD(31,1023) the lowest (15.26 Mb/s). As reported in Table 3, the differences in throughput among the schemes were substantially decreased in SCN_4, with those of OBO_CTRL and UORA_STD(15,255) being not much different from that of OPT_OCW. The interesting result was that the average throughput of UORA_STD(7,31) was close to that of UORA_STD(31,1023), which was not the case for SCN_3 or the static scenario (Figure 3). We repeated the simulation for SCN_5 where $N_{sta}^{o}$ was smaller than in SCN_3 and SCN_4. While UORA_STD(7,31), OPT_OCW, and OBO_CTRL achieved similar throughput values of around 17.1–17.8 Mb/s, UORA_STD(31, 1023) had the lowest throughput (13.3 Mb/s).

We focused on the throughput variation in the dynamic network environment, which can be evaluated with the value of Δ in Table 3. UORA_STD(31,1023) showed the largest variation in throughput for most cases. Three UORA_STD schemes had quite different throughput variations depending on the simulation scenario (e.g., Δ of UORA_STD(7,31) in SCN_4 was higher than that in SCN_3 by more than four times). In contrast, the throughput levels of OPT_OCW and OBO_CTRL in SCN_3 – SCN_5 were much smaller than in the other UORA_STD schemes.

### 5.3. The Effect of OBO Control Parameters

#### 5.3.1. The Effect of δ on Throughput and Fairness

We evaluate the effect of δ, the key parameter of OBO_CTRL used to adjust the OBO counter (see (6)). If δ has a large value, the OBO counter changes greatly, resulting in a fast response to changes in network conditions. However, a large value of δ is not desirable for stable OBO control.

**Figure 6:**
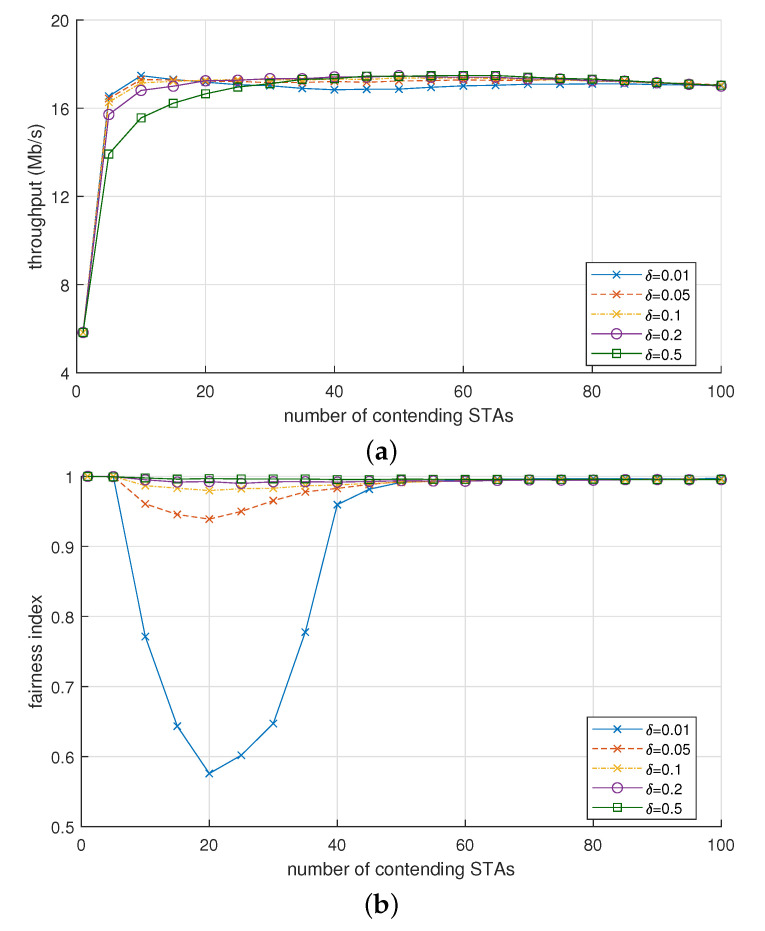
Effect of δ in OBO_CTRL on (**a**) throughput and (**b**) fairness.

Figure 6a shows the throughput of OBO_CTRL with various δ values ranging from 0.01 to 0.5 ($\alpha_{min}$ and $\alpha_{max}$ were fixed to 0.1 and 2.0, respectively). When $N_{sta}>25$, the effect of δ on the throughput of OBO_CTRL was marginal; the throughput was between 16.84 and 17.49 for the entire range of δ. However, when $N_{sta}\leq$ 20, the smaller value of δ increased the throughput (e.g., when $N_{sta}$ = 10, the throughput was 17.5, 16.8, and 15.6 Mb/s with δ = 0.01, 0.1, and 0.5, respectively).

In contrast to the throughput, δ had an obvious effect on the fairness. Figure 6b shows the value of Jain’s fairness index [30] calculated as

$$\frac{{\left(\sum_{i=1}^{N_{sta}}th_{i}\right)}^{2}}{N_{sta}\sum_{i=1}^{N_{sta}}th_{i}^{2}},$$

where $th_{i}$ is the throughput achieved by STA *i*. The fairness index has the maximum value of one when all the STAs have the same throughput, whereas its minimum value is $1/N_{sta}$ when only one STA monopolizes the whole network resource, i.e., $th_{i}>0$ and $th_{j}=0,\forall j\neq i$. As long as δ≥ 0.1, the fairness index was quite close to the ideal value of 1 for the entire range of $N_{sta}$. Similarly, as long as $N_{sta}\geq$ 50, the fairness index hardly changed from 1 regardless of the value of δ. However, when δ< 0.1 and $N_{sta}<$ 40, the fairness index was greatly deteriorated or affected by the values of δ and $N_{sta}$. For example, when δ = 0.01, the fairness index decreased below 0.6 as $N_{sta}$ increased up to 20, but it rather increased when $N_{sta}$ exceeded 20. In the $N_{sta}$ range from 10 to 35, a higher δ significantly increased the fairness index.

**Figure 7:**
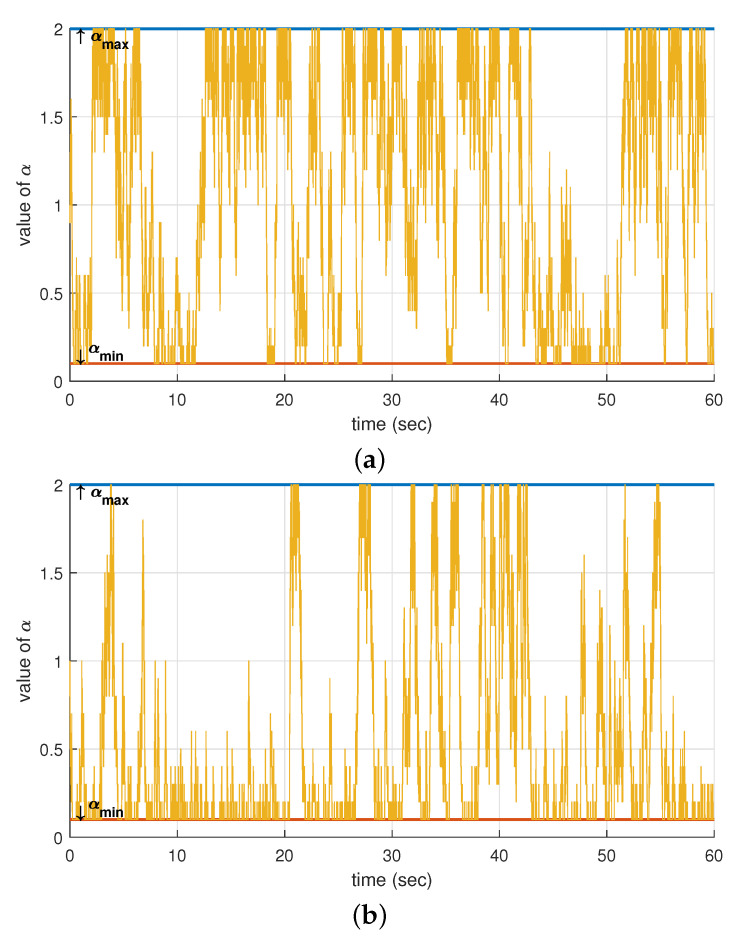

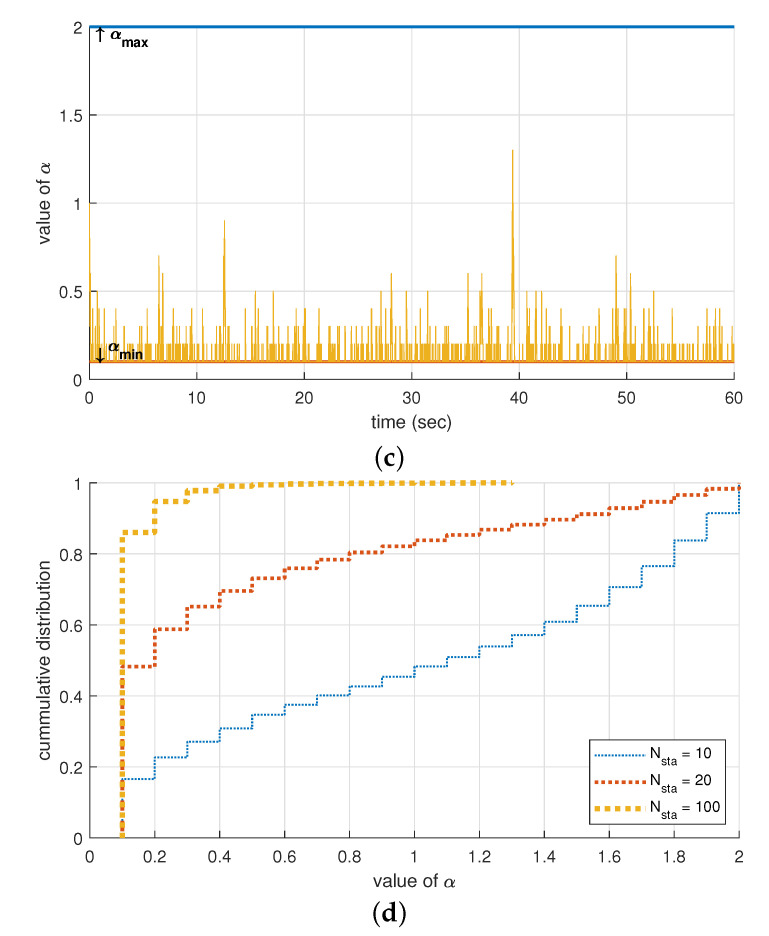
Observation of α when the number of contending STAs was (**a**) 10, (**b**) 20, and (**c**) 100, and (**d**) the cumulative distribution of α.

We investigated why the per-STA fairness of OBO_CTRL had been deteriorated by a certain configuration of δ and $N_{sta}$. For this purpose, we observed how α changes over time. Figure 7a–c shows changes in α during the simulation time (60 s) when $N_{sta}$ = 10, 20, and 100, respectively. From these observations, we discovered that the value of α notably changed between its minimum and maximum values ($\alpha_{min}$ = 0.1, $\alpha_{max}$ = 2) when $N_{sta}$ was small, but the fluctuation of α significantly decreased and was maintained at around its minimum value when $N_{sta}$ was large. Figure 7d shows the cumulative distribution of α measured in these simulations. When $N_{sta}$ = 10, the probability that α remains at $\alpha_{min}$ was 17%, but this changed to 48% and 86% for $N_{sta}$ = 20 and 100, respectively. From these results, we can infer the following. If $N_{sta}$ is small, each STA can have quite different values of α, and thus the deviation in channel access probability between the STAs increases. In this situation, a larger value of δ decreased the deviation in α between the STAs, whereas a smaller one changed α slowly, thereby maintaining a large deviation over a longer time. Consequently, the per-STA throughput could be quite different for each STA, which leads to a lower fairness index value.

In conclusion, the value of δ in our proposed scheme should be carefully set so as not to compromise fairness while improving throughput. We set the value of δ as 0.1 to satisfy this requirement, which provides nearly consistent performance in terms of throughput and fairness regardless of $N_{sta}$.

#### 5.3.2. The Effect of $\alpha_{min}$ and $\alpha_{max}$ on Throughput

Similar to δ, we investigated the effect of parameters $\alpha_{min}$ and $\alpha_{max}$ (i.e., the lower and upper bounds of α in OBO_CTRL).

**Figure 8:**
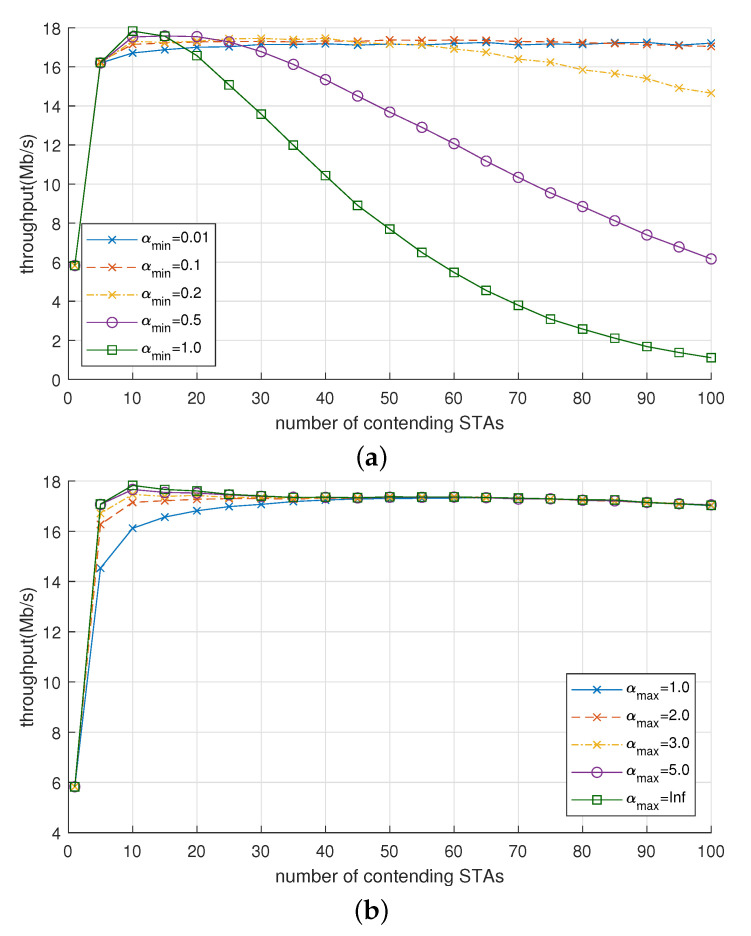
Effect of (**a**) $\alpha_{min}$ and (**b**) $\alpha_{min}$ on the throughput of OBO_CTRL.

First, we observed the effect of $\alpha_{min}$ and $\alpha_{max}$ on throughput. Figure 8a shows the throughput of OBO_CTRL with various values of $\alpha_{min}$ ranging between 0.01 and 1.0. (δ and $\alpha_{max}$ were fixed to 0.1 and 2.0, respectively). When $N_{sta}$ was small (≤ 20), $\alpha_{min}$ hardly affected the throughput. Moreover, when $N_{sta}$ was large (≥ 50), the throughput also changed little as long as $\alpha_{min}\leq$ 0.1 but rapidly decreased as $\alpha_{min}$ exceeded 0.1. For example, when $N_{sta}$ = 50, throughput started to decrease from 17.2 to 7.69 Mb/s as $\alpha_{min}$ increased from 0.2 to 1.0. It is important to note that when $N_{sta}$ is large, the larger value of $\alpha_{min}$ suppresses the channel access less in the proposed scheme, and so the collision probability increases and the throughput decreases accordingly.

Figure 8b shows the effect of $\alpha_{max}$ on throughput, where $\alpha_{max}$ ranges from 1.0 to *∞*. Note that the case where $\alpha_{max}=\infty$ means that α can increase without any upper bound. Here, both δ and $\alpha_{min}$ were fixed to 0.1. As opposed to $\alpha_{min}$ (see Figure 8a), $\alpha_{max}$ did not remarkably affect the throughput of OBO_CTRL. Especially when $N_{sta}\geq$ 50, the differences in throughput for the whole values of $\alpha_{max}$ were at most 0.1 Mb/s. When $N_{sta}\leq$ 20, the increase in $\alpha_{max}$ somewhat increased the throughput. By comparing the results in Figure 8, we can conclude that a small value of $\alpha_{min}$ (around 0.1) is desirable to achieve high throughput with OBO_CTRL, whereas $\alpha_{max}$ does not have a critical effect on the throughput.

#### 5.3.3. The Effect of $\alpha_{min}$ and $\alpha_{max}$ on Fairness

Table 4 lists fairness index values of OBO_CTRL with various values of $\alpha_{min}$, $\alpha_{max}$, and $N_{sta}$, from which we can make the following points:

- The fairness index value was mostly close to 1 and tended to increase when (i) $\alpha_{min}$ was large and $N_{sta}$ was small or (ii) $\alpha_{max}$ was small and $N_{sta}$ was large.
- The per-STA throughput fairness was degraded, and the fairness index was smaller than 0.9 when (i) $\alpha_{min}$ was small (0.01) and $N_{sta}$ was large (50–100) or (ii) $\alpha_{max}$ was infinite and $N_{sta}$ was small (10–20), implying the necessity of setting the upper bound for α.
- The fairness index was greater than 0.99 when (i) $\alpha_{min}$ was between 0.2 and 0.5 (regardless of $N_{sta}$) or (ii) $N_{sta}$ was between 50 and 100 (regardless of $\alpha_{max}$).

The reason for poor fairness index values under specific settings of $\alpha_{min}$ and $\alpha_{max}$ can be explained as follows. When $\alpha_{min}$ is small or $\alpha_{max}$ is large, the range of α increases accordingly. Subsequently, it is possible for some STAs to maintain a large value of α whereas others maintain a small one. Deviations in channel access probability per STA can be large, while per-STA throughput can be accordingly different. Last, by combining the results in Figure 8 and Table 4, we can find an important trade-off between throughput and fairness by setting the values of $\alpha_{min}$ and $\alpha_{max}$. A large $\alpha_{min}$ (close to 1) is not desirable to maintain high throughput, but it is helpful to improve fairness, while a small $\alpha_{max}$ can improve fairness at the cost of decreasing throughput.

### 5.4. The Effect of Varying the Number of RA-RUs

**Figure 9:**
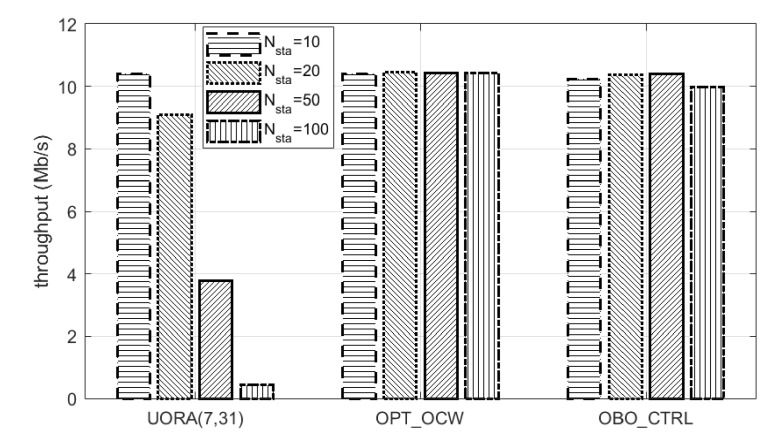
Throughput comparison when the number of RA-RUs was varied.

Up to now, we fixed the number of RA-RUs with AID 0 ($M_{ru}$) to 8. In this simulation, we observed and compared the performance of the UORA schemes when $M_{ru}$ was varied. Figure 9 shows a comparison of the throughput of UORA_STD(7,31), OPT_OCW, and OBO_CTRL when $M_{ru}$ was a random value uniformly distributed between 1 and 8 and each TF indicated this as the available number of RA-RUs. Recall that OBO_CTRL is completely identical to UORA_STD(7,31) if α of OBO_CTRL is not controlled and fixed at 1. The throughput of UORA_STD(7,31) rapidly decreased from 10.4 to 0.45 Mb/s as $N_{sta}$ increased from 10 to 100. However, OBO_CTRL maintained almost constant throughput regardless of $N_{sta}$, with the difference in the maximum and minimum throughputs of OBO_CTRL for all values of $N_{sta}$ being only 0.4 Mb/s. Its average throughput was 10.25 Mb/s, which is around 1.73 times that of UORA_STD(7,31) and almost equal to that of OPT_OCW. Another important point is that the change in $M_{ru}$ hardly affected the throughput per RU in OBO_CTRL. When $M_{ru}$ was fixed to 8, the average throughput of OBO_CTRL with $N_{sta}$ = 10 – 100 was 17.26 (Figure 3), and thus, the average throughput per RU was 2.16. Considering that the average value of $M_{ru}$ in this simulation was 4.5, the average throughput per RU was 2.28, which is close to the case when $M_{ru}$ is fixed. In summary, by simply adjusting the value of α, OBO_CTRL significantly increases the throughput, and its outstanding performance is maintained regardless of the value of or change in $M_{ru}$.

## 6. Conclusions and Future Work

We proposed a simple OBO control scheme to improve the throughput of UORA. In our proposed scheme, each STA controls its OBO counter in a distributed way based on the transmission result, so it does not require a signaling mechanism between the AP and the STA and is free from a signaling overhead. Moreover, the proposed mechanism works without any information about the number of contending STAs. The key point is to introduce a self-tunable parameter for determining the OBO counter. In this way, the STA accesses the RU in an aggressive manner to effectively decrease idle RUs when the number of contending STAs is small. At the same time, the STA accesses the RU in a conservative manner to avoid frequent collisions when the number of contending STAs is large. The extensive simulation results confirm that the slight and simple modification in the proposed mechanism results in a drastic throughput enhancement compared to the standard mechanism for IEEE 802.11ax and that the performance of our mechanism is very close to the ideally optimal mechanism that cannot be implemented practically.

In future work, we will devise a more efficient UORA mechanism by elaborating the OBO control rule of the proposed mechanism. We also plan to apply a reinforcement learning technology to improve the performance of UORA in the realistic environments of WLAN systems.

## Figures and Tables

**Table 1:** Simulation parameters.

Parameter	Value
Simulation time	60 s
$\left(OCW_{min},OCW_{max}\right)$	(7,31), (15,255), (31,1023)
Channel bandwidth	20 MHz
Guard interval	1.6 μs
OFDM symbol duration	12.8 μs
Number of subcarriers per RU	26
Number of RUs (AID=0)	8
Number of RUs (AID=2045)	1
Number of contending STAs	1∼100
Modulation and coding rate	64-QAM, 2/3
Data rate per RU	6.67 Mb/s
Slot time	9 μs
SIFS	16 μs
PHY header length	40 μs
Trigger frame length	100 μs
MU-BACK length	68 μs
Association request frame	38 bytes
MPDU	2000 bytes

**Table 2:** Simulation configurations for dynamic network scenarios.

Scenario no.	$N_{\mathrm{sta}}^{\mathrm{IN}}$	$N_{\mathrm{sta}}^{\mathrm{OUT}}$	$T_{c}$	$N_{\mathrm{sta}}^{o}$
SCN_1	2	0	4.0 s	1
SCN_2	0	2	4.0 s	50
SCN_3	8	8	1.25 s	100
SCN_4	8	8	1.25 s	20
SCN_5	8	8	1.25 s	10

**Table 3:** The average throughput and the difference between the 95th and 5th percentiles of the throughput of the UORA schemes under dynamic network scenarios 3–5.

Schemes	Average (Mb/s)			Difference (Δ) (Mb/s)		
	**SCN_3**	**SCN_4**	**SCN_5**	**SCN_3**	**SCN_4**	**SCN_5**
UORA_STD(7,31)	0.91	15.36	17.40	0.57	2.66	1.79
UORA_STD(15,255)	15.38	16.99	15.68	1.17	1.72	2.29
UORA_STD(31,1023)	17.38	15.26	13.28	1.36	2.29	2.88
OPT_OCW	17.29	17.48	17.84	1.33	1.30	1.49
OBO_CTRL	16.89	17.20	17.07	1.28	1.66	1.85

**Table 4:** Fairness index values for various values of $\alpha_{min}$s, $\alpha_{max}$s, and $N_{sta}$s.

$N_{\mathrm{sta}}$	$\alpha_{\min}$					$\alpha_{\max}$				
	**0.01**	**0.1**	**0.2**	**0.5**	**1**	**1**	**2**	**3**	**5**	**∞**
10	0.917	0.987	0.997	0.997	1.000	0.996	0.987	0.979	0.910	0.744
20	0.863	0.908	0.995	1.000	1.000	0.993	0.980	0.958	0.928	0.763
50	0.844	0.991	0.998	0.999	0.998	0.994	0.991	0.991	0.992	0.992
100	0.813	0.995	0.996	0.996	0.981	0.995	0.995	0.995	0.995	0.996

## Data Availability

Not applicable.
